# Task Autonomy of a Flexible Endoscopic System for Laser-Assisted Surgery

**DOI:** 10.34133/2022/9759504

**Published:** 2022-08-24

**Authors:** Hangjie Mo, Xiaojian Li, Bo Ouyang, Ge Fang, Yuanjun Jia

**Affiliations:** ^1^ Hong Kong Centre for Cerebro-Cardiovascular Health Engineering, Hong KongChina; ^2^ School of Management, Hefei University of Technology, Hefei, China; ^3^ Department of Mechanical Engineering, The University of Hong Kong, Hong Kong, China; ^4^ Department of Automation, University of Science and Technology of China, Hefei, China

## Abstract

Laser beam steering has been widely studied for the automation of surgery. Currently, flexible instruments for laser surgery are operated entirely by surgeons, which keeps the automation of endoluminal surgery at the initial level. This paper introduces the design of a new workflow that enables the task autonomy of laser-assisted surgery in constrained environments such as the gastrointestinal (GI) tract with a flexible continuum robotic system. Unlike current, laser steering systems driven by piezoelectric require the use of high voltage and are risky. This paper describes a tendon-driven 2 mm diameter flexible manipulator integrated with an endoscope to steer the laser beam. By separating its motion from the total endoscopic system, the designed flexible manipulator can automatically manipulate the laser beam. After the surgical site is searched by the surgeon with a master/slave control, a population-based model-free control method is applied for the flexible manipulator to achieve accurate laser beam steering while overcoming the noise from the visual feedback and disturbances from environment during operation. Simulations and experiments are performed with the system and control methods to demonstrate the proposed framework in a simulated constrained environment.

## 1. Introduction

Laser-assisted surgery has the advantages of effective hemostasis and minimization of surrounding tissue damage, and thus, it has received extensive attention [1–3]. However, the use of lasers as a surgical tool faces challenging problems in the transmission of high-power laser energy and the precise control of the laser beam [4]. Currently, fiber-free and fiber-based technologies are mainly used for laser-assisted surgery [5–6]. Fiber-free technology uses a scanning mirror mounted on the microscope to control the laser beam and direct it to the target location. However, large-sized scanning mirrors and microscopes are placed outside the patient’s body, resulting in a long distance between the laser manipulation system and the surgical site. This limits the ability to deploy a laser beam in the direct path from the microscope-mounted scanning mirror to the surgical site in a constrained environment. Fiber-based technologies use optical fibers or waveguides to transfer laser energy from the laser source to the surgical site [7]. Considering the flexibility of the optical fiber, this transmission system can allow access to difficult-to-access surgical sites and improve operation accuracy compared to fiber-free laser systems. For example, an optical fiber has been connected to a da Vinci surgical tool (Intuitive Surgical, Inc., USA) to remove supraglottic cancer and oropharyngeal cancer with laser energy [8]. In addition, a bending manipulator for intrauterine fetal surgery was developed, and its feasibility was analyzed through in vitro and in vivo experiments [9]. However, these systems still require surgeons to operate the instruments step by step, and surgeons must address many challenges, such as overlearning, operational difficulties, and physical and mental fatigue. Therefore, there is a need for a more automated method to reduce the burden on surgeons. Considering the complexity and diversity of surgical tasks, it is currently not possible to fully automate the entire surgical procedure, but it is feasible to automate certain individual tasks guided by surgeons.

By combining with computer-aided control, endoscopic laser-assisted surgery technology has recently been developed to achieve robot-assisted laser surgery [6]. For example, a compact endoscopic system is developed to achieve robot-assisted vocal cord laser microsurgery. Microscanning mirrors with high scanning range based on MEMS technology are assembled at the tip of the endoscopic system to steer the laser beam [10]. The interface between the surgeon and the robot has also been developed to achieve robot-assisted surgery to reduce the burden on the surgeon. Similarly, a microactuator system is designed to achieve a wide range of laser beam steering. Two mirrors are driven by linear actuators, which convert linear motion into rotational motion of the mirrors through crank-slider transmission [11]. However, both systems are driven by piezoelectric motors, which require the use of high voltage and are risky. Tendon-driven flexible manipulators with the characteristics of flexibility and dexterity provide a feasible solution to steer the laser beam [12]. However, it is challenging to integrate a flexible manipulator into an existing endoscopic system while controlling the flexible manipulator to steer the laser beam accurately. This is because the shape of the endoscopic system will affect the movement of the manipulator, and since the shape of the endoscopic system is unknown, it is difficult to compensate for this effect.

In this paper, we proposed a workflow that enables task autonomy for laser-assisted treatment by using a flexible robotic system fiber-based technologies to reduce the burden on surgeons while achieving accurate laser beam steering. We first developed a flexible robotic system with a diameter of 12 mm, which includes a flexible manipulator with a diameter of 2 mm inside the system. The endoscope has three degrees of freedom (DOF), including 2-axis bending and translation, and a manipulator with translation, rotation, and bending DOF. An optical fiber is installed inside the manipulator to achieve laser transmission. In order to achieve automatic laser beam steering, the movement of the flexible manipulator is decoupled from that of the endoscopic system through certain mechanical structures and a new cable routing method. Second, a master/slave control method guided by surgeons is applied to control the endoscope into the natural orifice and search the surgical site, and a new population-based model-free control method is applied to the flexible manipulator to achieve automatic laser beam steering. In contrast to current model-free methods in controlling flexible manipulator [13–15], the proposed model-free method for the first time considers the influence of noise from the endoscope and achieve a robust visual servo control strategy.

## 2. Materials and Methods

### 2.1. Workflow of Laser-Assisted Therapy

**Figure 1:**
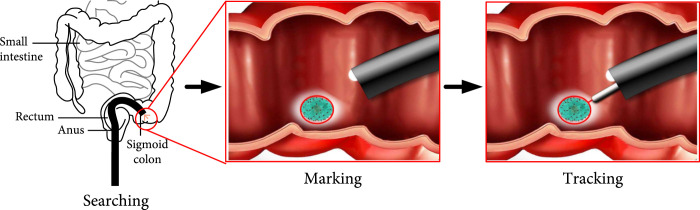
Workflow of task autonomy for laser-assisted endoscopic surgery.

In this paper, we consider laser-assisted endoscopic submucosal dissection (ESD) as a potential surgical procedure. The ESD procedure is described below [16]. The margin of the lesion is first identified and then marked outside the margin. After marking, a mixture of sodium hyaluronate is injected into the lesion to visualize the target area. Submucosa incisions are then made around the lesion by laser. Therefore, ESD is time-consuming and involves steps such as marking, injection, incision, and dissection. Figure 1 shows the workflow to complete this surgical task. First, the surgeon uses the console to control the endoscope to search for the surgical site. The desired path is then generated based on the surgeon. The robot can then automate the tissue dissection by using a control algorithm to manipulate the laser spot to follow the defined trajectory.

### 2.2. Mechanical System

**Figure 2:**
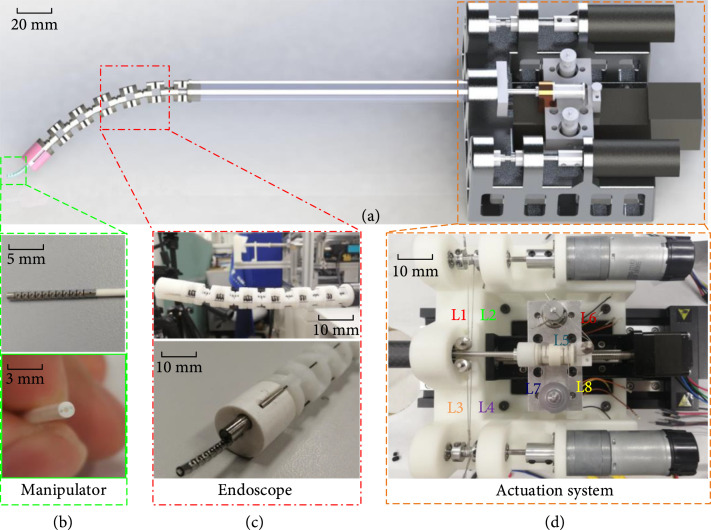
Schematic of the designed endoscopic system. (a) CAD model showing the key components of the designed flexible robotic endoscope system. (b) The flexible manipulator (2.0 mm) and the multilumen flexible tube. (c) The endoscope integrates a camera (3.0 mm) and the manipulator. (d) The actuation system showing the arrangement of cables and motors. The degrees of freedom of both the endoscope and the manipulator are three.

In the proposed workflow, several issues are considered in the design of a compact endoscopic system. First, considering the anatomical constraints of the colon and rectum, the total diameter of the instrument must be limited to 20 mm [17]. In our study, we designed the instrument with a diameter of 12 mm to meet this requirement. Second, a flexible manipulator is needed to transmit and control the laser beam from the laser source to the target position on one channel, and a camera on another channel is required for visual feedback. Third, when the endoscope is introduced to the surgical site via a natural orifice, its shape will change, which affects the kinematics of the manipulator, so it needs to be compensated [18]. To simplify the control algorithm, the motion of the overall endoscopic system and that of the manipulator inside the endoscopic system must be decoupled. Figure 2 shows a schematic of the designed endoscopic system.

#### 2.2.1. Endoscope

**Figure 3:**
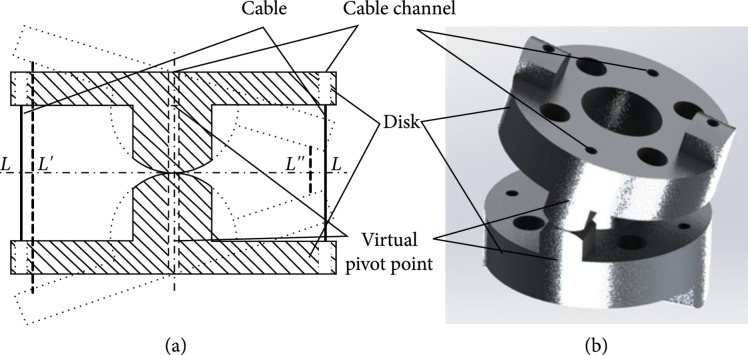
Theory of cable-balancing pivotal mechanism. (a) Schematic of the cable-balancing pivotal mechanism. (b) CAD model of the designed disks.

The endoscope employs a continuum robot structure and is composed of multiple serial virtual pivot disks with advantages of low friction, symmetric actuation, and large force transmission [19–21]. As shown in Figure 3(a), two disks with circular surfaces come into contact with each other at a rolling contact point. The relative rotation between the disks is achieved by pulling cables. Upon rotation of the disks, the cables shift to new positions, as shown by the dashed lines. Each pair of cables is kept equidistant from the centerline, and the elongation of the cable length on one side is equal to the amount of shortening of the cable length on the other side, such that

(1)L′−L=L−L″,

where L is the original length of the cables, $L^{\prime }$ is the length of the elongated cable, and $L^{\prime \prime }$ is the length of the shortened cable. This cable balancing feature ensures minimal backlash for each pair of cables. For each joint, since the centerline is the line connecting the two virtual pivot points, the length of the centerline is equal to the sum of the radii of the two circles and is unchanged. Thus, the total length of the endoscope does not change.

#### 2.2.2. Manipulator

**Figure 4:**
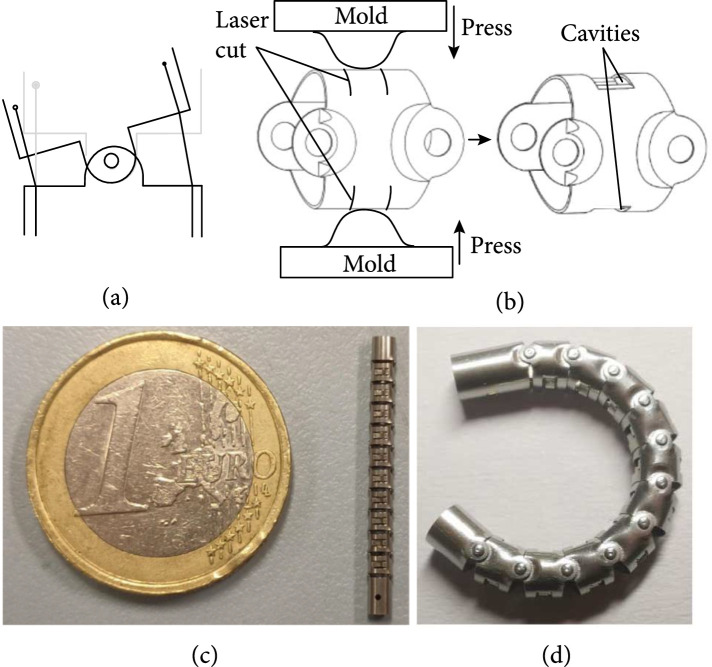
Structure of the manipulator. (a) Rolling joint. (b) Machining process. (c) Prototype. (d) Detail of the rolling joint.

**Figure 5:**
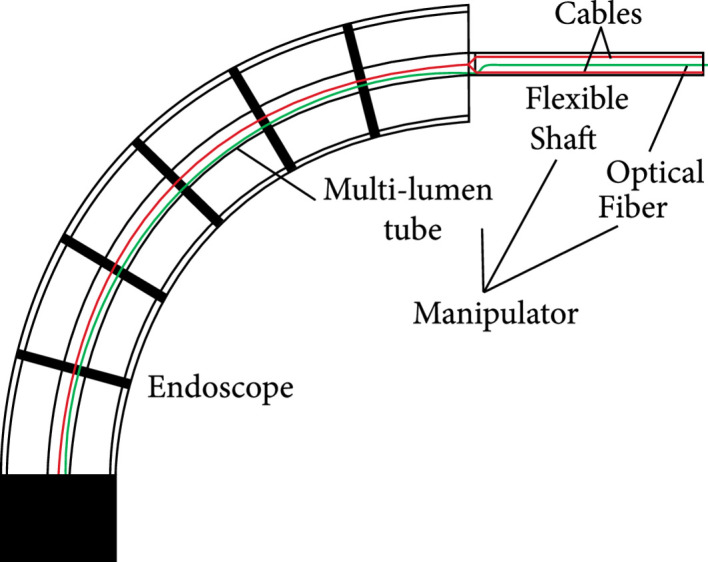
Schematic of motion decoupling between the endoscope and manipulator.

The manipulator consists of a flexible shaft and a multilumen tube, with a diameter of 2 mm. As shown in Figure 4(a), the rotary joint of the flexible shaft is a pivot hinge realized by riveting. With a given wall thickness, the cavities for cables can be obtained by the following methods. As shown in Figure 4(b), on the surface of each disk, four slots are cut by using laser processing technology. A multilumen flexible tube (thermoplastic polyurethanes) is attached to the flexible shaft. The two cables first pass through these two cavities of the flexible shaft and then routed to the central lumen of the multitube. The optical fiber passes through the lumen on one side of the tube to the tip of the manipulator, as shown in Figure 5.

#### 2.2.3. Actuation Mechanism

**Figure 6:**
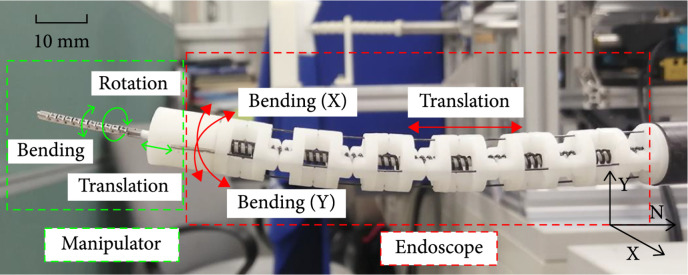
Movement of the endoscope and the manipulator.

As shown in Figure 6, the x-axis is parallel to the ground plane whereas the y-axis is perpendicular to it. The endoscope is driven by four evenly distributed cables labeled L1, L2, L3, and L4, as shown in Figure 2(d). The endoscope can be bent around the y-axis by pulling L1 and L2 and bent around the x-axis by pulling L3 and L4. Linear motion is achieved by a linear guide. For the manipulator, we can bend on one plane by pulling cables labeled L5 and L6. The motion of self-rotation is achieved by pulling cables labeled L7 and L8. A linear rail is also applied to realize the linear motion.

#### 2.2.4. Kinematic Decoupling

Figure 5 shows that the manipulator including a flexible shaft and a multilumen tube is on the central channel of the endoscope. The shape of the tube is controlled by the endoscope, while the bending of the flexible shaft is controlled by pulling two cables. These two cables first pass through the two cavities of the flexible shaft and then routed to the central lumen of the multitube. Since the length of the centerline remains constant regardless of how the endoscope is bent, and both cables are maintained at the centerline, the length of the cables will not be affected by the bending of the endoscope. As a result, the movements of the endoscope and the manipulator are decoupled. If the cables are placed in two lumens on sides of the tube, as shown in Figure 2(a), the endoscope’s bending will cause the cables to be shortened on one side and lengthened on the other side. Two independent actuators and a compensation algorithm are needed to accommodate these length changes to keep the cables’ tension.

### 2.3. Control

#### 2.3.1. Master/Slave Control for Endoscope

**Figure 7:**
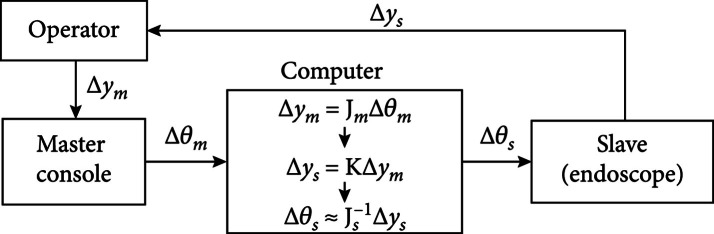
Master/slave control diagram.

When introducing an endoscope into a surgical site through a natural orifice, safety and reliability are the primary considerations. Therefore, a master/slave control method that is fully supervised and controlled by the surgeon is applied in this step [22, 23]. The surgeon operates the master console based on the endoscopic view at the master site and sends the command to the slave devices. At the slave site, this command based on the surgeon’s movement will be converted to the endoscope’s actuators. Figure 7 shows the control diagram.

The mapping from the master console to the slave endoscope is expressed as follows:

(2)ym=fθm,(3)ys=gθs,

where $f\left(\right)$ and $g\left(\right)$ are the mapping function; $y_{m}$ and $y_{s}$ are the positions of the end-effector of the master console and slave endoscope, respectively; and $\theta_{m}$ and $\theta_{s}$ are the joint signals of the master console and the slave endoscope, respectively. Differentiating equation (2) with respect to time t yields a linear function:

(4)y˙m=Jmθ˙m,y˙s=Jsθ˙s,

where $J_{m}\in {\mathbb{R}}^{n\times n}$ and $J_{s}\in {\mathbb{R}}^{n\times n}$ are the Jacobian matrices of master console and slave endoscope [24], respectively. A simple controller is utilized to compute the position ${\Delta y}_{s}$ of the slave endoscope, which is expressed as follows:

(5)Δys=KΔym,K=k1⋯0⋮⋱⋮0⋯kn∈Rn×n,

where K is a diagonal matrix. The joint signal ${\Delta \theta }_{s}$ of the endoscope can be calculated with the inverse Jacobian matrix ${J_{s}}^{-1}$ of the slave endoscope.

#### 2.3.2. Model-Free Control for Manipulator

After the trajectory is defined by a surgeon, the manipulator is controlled to steer the laser beam to follow the given trajectory with visual feedback. A control approach that circumvents complex modeling and derives kinematics from the sensing data provides a feasible solution to this problem [25–28]. A model-less method was proposed to control a continuum robot working in a constrained environment [29]. In our study, we propose the use of a population-based method [30] for model-free visual servoing control. This control method approximates the system based on the secant model by using more than two previous iterates, in order to enhance robustness to the image noise when the robots work in the lighting environment of low luminance.

A function $H\left(\right)$ that maps from the actuation space to the image space can be expressed as follows:

(6)y=Hθ,

where $y=\left[y_{1}\cdots y_{k}\right]$ denotes the image feature vector, $\theta =\left[\theta_{1}\cdots \theta_{k}\right]$ is the joint signal, and k is the number of DOF. Differentiating equation (6) with respect to time t yields a nonlinear transfer function as follows:

(7)y˙=H˙θ.

The nonlinear transfer function $\dot{H}\left(\right)$ can be approximated by a secant model in a local region of θ, expressed as

(8)y˙=Jtθ˙,

where $J\left(t\right)\in R^{k\times k}$ is the Jacobian matrix, which is the very research focus of visual servoing. At each discrete time step t, $J\left(t\right)$ can be presented as $J_{t}$ for simplicity.

The initial Jacobian matrix is first obtained by moving each actuator of the manipulator with an incremental joint signal $\Delta \theta_{i}$ and measuring the change of the image feature ${\Delta y}_{i}$, which is

(9)J0=Δy1Δθ1⋯ΔylΔθl.

To ensure that the secant model [8] is as close as possible to the nonlinear transfer function [7], the Jacobian matrix $J_{t}$ can be updated in the least squares sense as follows, which depends on a finite population of previous iterates:

(10)Jt+1=argminJtSt IΩ2OOΓ2−YtJtΩ2OOΓ2F2.

Equation (10) yields that

(11)Jt+1=Jt+Yt−JtStΩ2StTΓ2+StΩ2StT−1,

where $J_{t+1}$ is the estimated Jacobian matrix at the next time step t+1; $S_{t}$ is a population of the past p joint signal changes, i.e., $S_{t}={\left[\begin{array}{cccc} {\Delta \theta }_{t-p+1} & {\Delta \theta }_{t-p+2} & \cdots & {\Delta \theta }_{t} \end{array}\right]}^{\text{T}}$; $Y_{t}$ is the population of the past p image feature changes, i.e., $Y_{t}={\left[\begin{array}{cccc} {\Delta y}_{t-p+1} & {\Delta y}_{t-p+2} & \cdots & {\Delta y}_{t} \end{array}\right]}^{\text{T}}$; $\Gamma^{2}$ is a matrix measuring the accuracy of initial estimation of the Jacobian matrix. Ω is the diagonal weight matrix, expressed as

(12)Ω2=diagω1ω2⋯ωp,ωi=λp−i+1ρi,ρi=σ2σ2+eiTei,

where 0<λ≤1 is a forgetting factor used to update the Jacobian matrix based on previous p updates in an exponentially decaying manner to lessen the weight of old data during the estimation process and $\rho_{i}$ is used to filter image feature with large error to improve robustness to noise. $\sigma =\text{med}\left(\left|\Delta y_{t-p+i}-\text{med}\left(Y_{t}\right)\right|\right)$ is a parameter based on the median absolute deviation to quantify the distribution of the probability distribution, and $e_{i}$ is the error between the actual image feature change and that estimated by the secant model.

With the estimated Jacobian matrix, a model predictive controller is applied to control the joint signal of the motor in the next time step [31–33]. The reference output position of the laser spot at time t+s is planned as follows:

(13)yrt+s=yd−e−ρsξt,

where s represents the prediction horizon, $y^{\text{d}}$ is the desired position, ρ is a positive constant, and $\xi \left(t\right)$ is the error between the desired and actual position. Input Δθ is assumed to remain constant from t to t+s. Error ε between the planned and the estimated position at time *t* + *s* can be calculated as follows:

(14)εt+s=1−e−ρsξt−sJtΔθ.

Input θ is then calculated by minimizing error ε over the period from t to t+h, expressed as

(15)min12∫0hαs1−βsξt−sJtΔθ2+ΔθTQΔθds,

where h is the total length of prediction horizon, 0<α≤1, $\beta =e^{-\rho },$ and Q is a symmetric and positive definite matrix. The control input is expressed as

(16) Δθ=mJt+hJtT+Q+n−pξtsubject tom=h2αh−2nlnα n=hαhlnα−αh+1ln2α p=hαβhlnαβ−αβh+1ln2αβ

## 3. Results

### 3.1. Simulations

**Figure 8:**
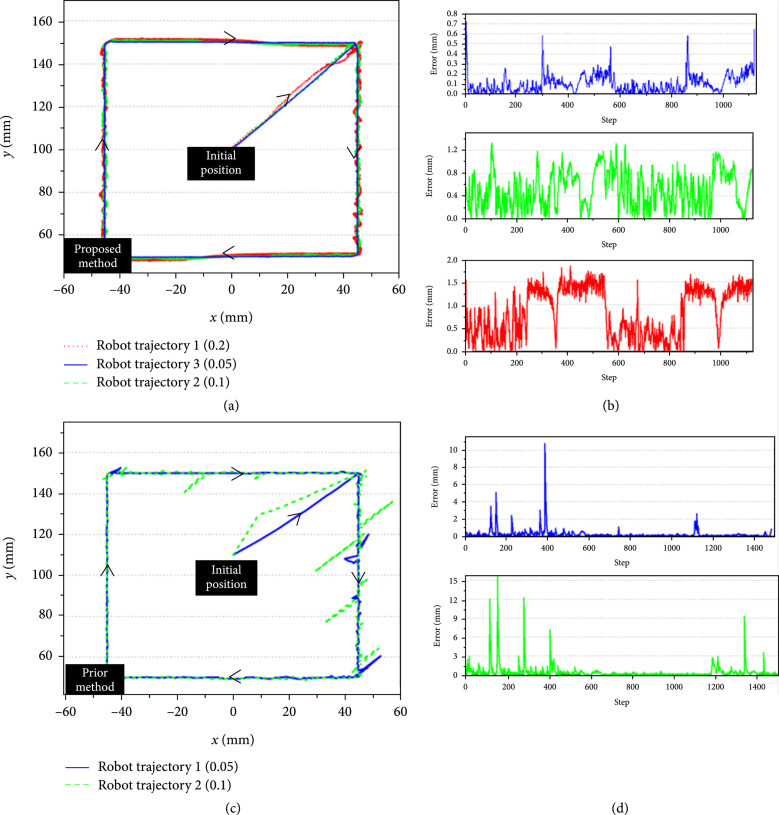
Comparison of trajectory tracking of manipulator with different noises.

Simulation was conducted in MATLAB to compare the performance of the proposed control method with previous method [15] in variance noise. A simple continuum robot model [34] was used in the simulation study, which is specified as follows:

(17)x=lθ11−cosθ1+w1,y=lθ1sinθ1+θ2+w1,

where l is the length of the manipulator, $\theta_{1}$ and $\theta_{2}$ are the inputs of the actuator, and $w_{1}$ is the noise drawn from Gaussian distribution with mean of zero and different variance (0.05, 0.1, and 0.2). Figure 8(a) shows that the tip of the manipulator follows the rectangular trajectory well under different noise levels. Figure 8(b) shows the tracking error with the defined noises. The deviations become larger at the corner of the rectangle when the noise with a variance of 0.05. The tracking error increases with the increase of noise, but the increase degree is not significant to affect the tracking result. Figure 8(c) shows the result of the previous control method [15]. Due to the presence of noise, many spikes appear during trajectory tracking. Figure 8(d) shows the corresponding tracking error. It is seen that the tracking error increases significantly with the increase of the noise level by using a previous method, while the proposed method can eliminate the influence of noise and track the trajectory with low error level.

### 3.2. Experiments

**Figure 9:**
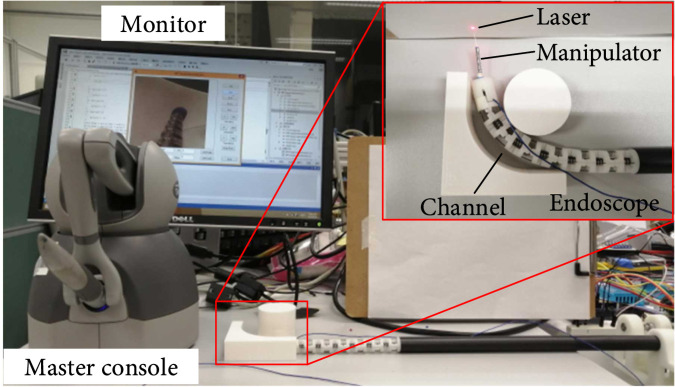
Experimental platform.

**Figure 10:**
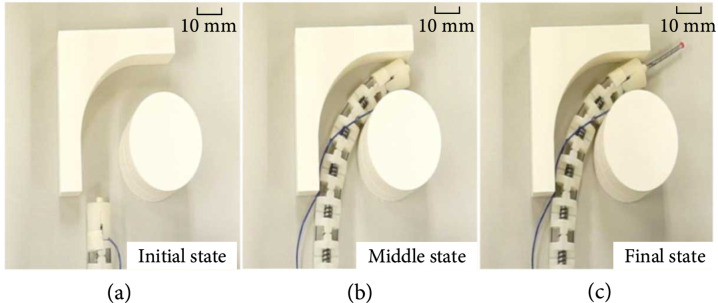
Experimental results of the master/slave control.

Experiments were performed on the designed endoscopic system to demonstrate the proposed workflow in a simulated constrained environment, as shown in Figure 9. 3D printing was used to manufacture small and nonsupporting parts, and mechanical machining was used to support and connect components. To limit the relative position of the disks when bending the endoscope, Ni-Ti memory alloy wires with a diameter of 0.5 mm were located within the bending joint part of the endoscope and then connected to steel wire ropes through crimping tubes. A camera (3.0 mm) including an LED light source and a CMOS of OV9734 (OmniVision Technologies, Inc.) was installed at the end effector of the endoscope. The laser spot was generated by a laser (30 mW) and transmitted via an optical fiber inside the manipulator. The master console is a Geomagic Touch (3D Systems, Inc.). Figure 10 illustrates the designed prototype system. The data streams of the master console and the camera occurred at frequencies of 1000 Hz and 30 Hz, respectively. The control loop ran at a frequency of 10 Hz (C++).

#### 3.2.1. Target Site Searching

The first experiment was conducted to control the endoscope to access a constrained environment and search the target site by using the master/slave control method. The movement of the master console controlled by an operator can be detected only when the button on the handle was pressed. The operator’s movement was sent to the actuators to move the endoscope. With the aid of the camera installed at the end effector, the video of the space can be shown on the screen to help the operator determine the target site. As shown in Figure 10(a), the endoscope was first placed outside of the constrained environment. It was then inserted into the constrained environment while interacting with the obstacles as presented in Figure 10(b). Figure 10(c) shows that the manipulator stretched out from the endoscope. It is seen that when the 2 mm manipulator stretched out from the bended endoscope, it remains straight, which proves the movement of the endoscope cannot affect the manipulator.

#### 3.2.2. Point Tracking

**Figure 11:**
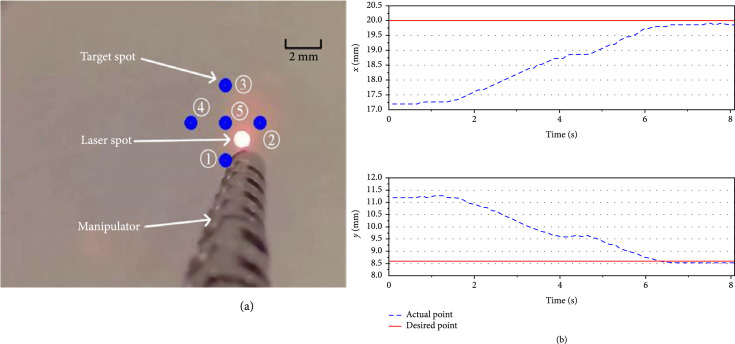
Target point tracking by controlling the manipulator.

In the second experiment, the laser spot was controlled to approach target points automatically by using the proposed model-free controller. The position of the laser spot was controlled through the control of the manipulator tip. When the operator identified the target site, the position of the endoscope was fixed, and multipoints were defined by the operator. As shown in Figure 11(a), after five points were defined, the center of the laser spot was detected with image processing by using the camera installed on the end effector. The laser spot was controlled to track the target points one by one by using the designed control method. Figure 11(b) shows that the laser spot has successfully converged from the first point [17.20, 11.20] to the second point [20.00, 8.75]. The steady-state error (less than 0.2 mm) is mainly caused by system error and estimation error.

#### 3.2.3. Trajectory Tracking

**Figure 12:**
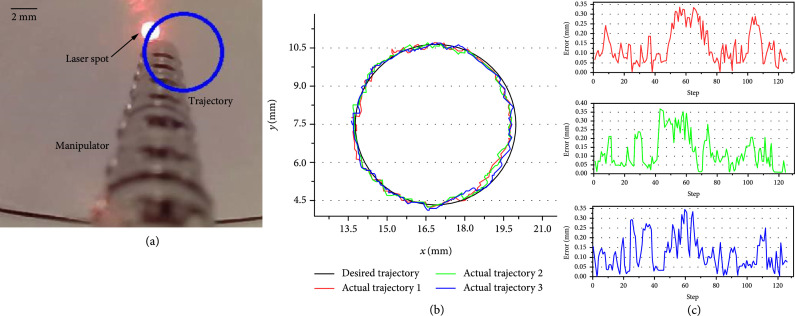
Results of the circle trajectory tracking. (a) Trajectory tracking. (b) Trajectory tracking errors of the three repeated tests.

**Figure 13:**
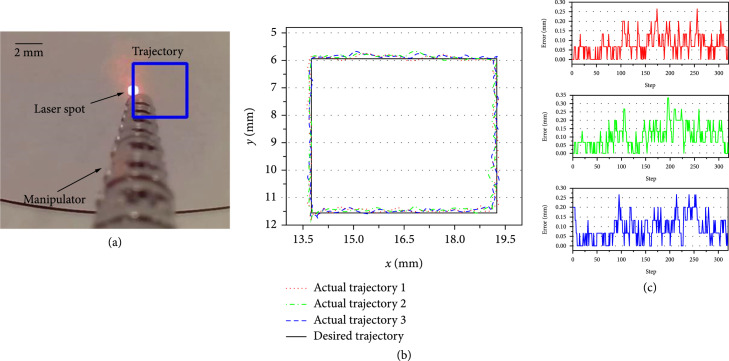
Results of the rectangular trajectory tracking. (a) Trajectory tracking. (b) Trajectory tracking errors of the three repeated tests.

In the third experiment, the laser spot was controlled to track a circle and rectangular trajectories three times. As shown in Figures 12 and 13, the circle and rectangular trajectories were defined first. The laser point was then controlled to repeatedly track the defined curve by using the proposed model-free control. Figures 12(b) and 13(b) show the trajectory tracking results, and Figures 12(c) and 13(c) show the tracking errors of the three repeated tracking experiments. The error was defined as the closest distance from the current position to the rectangle. Tables 1 and 2 provide a summary of the tracking errors in the three experiments. Note that the root mean square error (RMSE) is calculated here to further illustrate the trajectory tracking performance.

**Table 1:** Circle trajectory tracking error.

Experiment No.	Maximum error (mm)	RMSE (mm)
1	0.334	0.160
2	0.366	0.149
3	0.333	0.132

**Table 2:** Rectangular trajectory tracking error.

Experiment No.	Maximum error (mm)	RMSE (mm)
1	0.267	0.101
2	0.333	0.126
3	0.267	0.117

#### 3.2.4. Comparison of Trajectory Tracking

**Figure 14:**
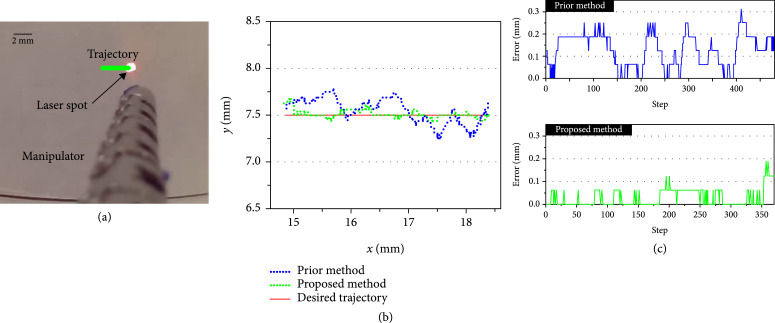
Comparison of trajectory tracking. (a) Trajectory tracking of laser spot. (b) Trajectory tracking results with the model-less control [15] and the proposed control method. (c) Trajectory tracking errors.

The last experiment was conducted to compare the trajectory tracking between the previous control method [15] and the proposed control method. As shown in Figure 14(b), the blue dot line is the trajectory of using the prior control method, and the green dot line is the trajectory of using the proposed control method. Figure 14(c) shows the corresponding tracking error. The maximum error of the proposed control method is 0.1875 mm, and the RMSE is 0.0395 mm, which is smaller than that of the prior control with a maximum error of 0.3125 mm and RMSE of 0.0751 mm. This experiment demonstrated the effectiveness of the proposed population-based control method.

## 4. Discussion

This paper presents a new workflow of laser-assisted endoscopic surgery in a constrained environment, which has the potential to achieve task autonomy in endoluminal surgeries, such as ESD with low cost. A robotic system is designed to achieve the workflow. The system includes an endoscope with a diameter of 12 mm and a tendon-driven flexible manipulator with a diameter of 2 mm, which is integrated into the endoscope, to manipulate a laser beam. The movement of the manipulator and that of the endoscope is decoupled through specific mechanical structures and newly designed cable routing. A master/slave control method is used to control the endoscope into the natural orifice and search the surgical site, and a population-based model-free control method is developed to control the manipulator to steer the laser beam automatically which eliminate the influence of noise. Successful experiments are performed to demonstrate the effectiveness of the workflow for laser-assisted endoscopic surgery in a confined environment. This study provides a useful solution for laser-assisted endoscopic surgery in a constrained environment. In future work, we will improve the system for specific surgical needs and assess the applicability to actual surgery through ex vivo and in vivo tissue ablation assessments.

## Data Availability

All data needed to evaluate the conclusions in the paper are present in the paper or in the Supplementary Materials.

## Supplementary Materials

Supplementary Materials Supplementary 1. The calculations process of the control input in equation (16) and the proof of stability. Supplementary 2. Movies S1: the content of the video is the four experimental results, which is used to evaluate the proposed workflow that enables task autonomy for laser-assisted treatment using flexible robots.
