# Supplementary material for: Task Autonomy of a Flexible Endoscopic System for Laser-Assisted Surgery
Source: Cyborg Bionic Syst. 2022 Aug 24;2022:9759504. doi: 10.34133/2022/9759504 (PMC11014730; doi:10.34133/2022/9759504)
Supplement: Supplementary Materials — Supplementary 1. The calculations process of the control input in equation (16) and the proof of stability. Supplementary 2. Movies S1: the content of the video is the four experimental results, which is used to evaluate the proposed workflow that enables task autonomy for laser-assisted treatment using flexible robots. [file 9759504.f1.zip › Supplementary 1.pdf]

Supplementary 1

Input $\boldsymbol{\theta}$ is calculated by minimizing error over the period from *t* to *t*+*h*, expressed as:

$$\min \frac{1}{2}(\int_0^h \alpha^s \left\|(1-\beta^s)\boldsymbol{\xi}(t) - s\mathbf{J}_t\Delta\boldsymbol{\theta}\right\|^2 + \Delta\boldsymbol{\theta}^{\mathrm{T}}\mathbf{Q}\Delta\boldsymbol{\theta}ds) \,, \tag{15}$$

Differentiating eq. (15) with respect to $\Delta\boldsymbol{\theta}$, gradient ∇ is calculated as follows:

$$\nabla = \int_0^h -s\alpha^s \mathbf{J}_t^{\mathrm{T}}((1-\beta^s)\boldsymbol{\xi}(t) - s\mathbf{J}_t\Delta\boldsymbol{\theta}) + \mathbf{Q}\Delta\boldsymbol{\theta}ds \,, \tag{16}$$

Control input $\Delta\boldsymbol{\theta}$ is derived by setting $\nabla = 0$.

$$h\mathbf{J}_t^{\mathrm{T}+}\mathbf{Q}\Delta\boldsymbol{\theta} + \int_0^h s^2\alpha^s ds \mathbf{J}_t\Delta\boldsymbol{\theta} = \int_0^h s\alpha^s\left(1-\beta^s\right)\boldsymbol{\xi}\left(t\right)ds \tag{17}$$

Then, the control input $\Delta\boldsymbol{\theta}$ can be expressed as:

$$\begin{aligned} &\Delta\boldsymbol{\theta} = \left(m\mathbf{J}_t + h\left(\mathbf{J}_t^{\mathrm{T}}\right)^+\mathbf{Q}\right)^+\left(n-p\right)\boldsymbol{\xi}(t) \\ \text{subject to} \quad & m = \int_0^h s^2\alpha^s ds = \left(h^2\alpha^h - 2n\right)\big/\ln\alpha \\ & n = \int_0^h s\alpha^s ds = \left(h\alpha^h\ln\alpha - \alpha^h + 1\right)\big/\ln^2\alpha \\ & p = \int_0^h s\left(\alpha\beta\right)^s ds = \left(h(\alpha\beta)^h\ln(\alpha\beta) - (\alpha\beta)^h + 1\right)\big/\ln^2(\alpha\beta) \end{aligned} \tag{18}$$

The error between the desired output $\mathbf{y}^{\mathrm{d}}$ and the actual output $\mathbf{y}(t)$ is defined as:

$$\boldsymbol{\xi}(t) = \mathbf{y}^{\mathrm{d}} - \mathbf{y}(t) \,, \tag{19}$$

where $\mathbf{y}^{\mathrm{d}}$ is the desired position, $\mathbf{y}(t)$ is the actual output.

Differentiating eq. (19) with respect to time and substituting the result into eq. (8) in the manuscript, we obtain

$$-\dot{\boldsymbol{\xi}}(t) = \mathbf{J}_t\Delta\boldsymbol{\theta} \,. \tag{20}$$

Substituting (18) into (20) yields the error dynamics as follows:

$$\left(m\mathbf{I} + h\mathbf{J}_t^{\mathrm{T}+}\mathbf{Q}\mathbf{J}_t^+\right)\dot{\boldsymbol{\xi}}(t) + \left(n-p\right)\boldsymbol{\xi}(t) = 0 \,. \tag{21}$$

As $m > 0$, $n-p > 0$, $h > 0$, and $\mathbf{J}^{\mathrm{T}+}\mathbf{Q}\mathbf{J}^+$ is a positive definite matrix, the controlled system is stable.
